# A Non-Uniformly Under-Sampled Blade Tip-Timing Signal Reconstruction Method for Blade Vibration Monitoring

**DOI:** 10.3390/s150202419

**Published:** 2015-01-22

**Authors:** Zheng Hu, Jun Lin, Zhong-Sheng Chen, Yong-Min Yang, Xue-Jun Li

**Affiliations:** 1 Science and Technology on Integrated Logistics Support Laboratory, National University of Defense Technology, Changsha 410073, China; E-Mails: zhenghu@nudt.edu.cn (Z.H.); czs_study@sina.com (Z.-S.C.); yangyongmin@163.com (Y-M.Y.); 2 Hunan Province Key Laboratory of Health Maintenance of Mechanical Equipment, Hunan University of Science and Technology, Xiangtan 411100, China; E-Mail: hnkjdxlxj@163.com

**Keywords:** blade tip-timing, non-uniformly sampled signal, under-sampled signal reconstruction, on-line vibration, band-pass sampling

## Abstract

High-speed blades are often prone to fatigue due to severe blade vibrations. In particular, synchronous vibrations can cause irreversible damages to the blade. Blade tip-timing methods (BTT) have become a promising way to monitor blade vibrations. However, synchronous vibrations are unsuitably monitored by uniform BTT sampling. Therefore, non-equally mounted probes have been used, which will result in the non-uniformity of the sampling signal. Since under-sampling is an intrinsic drawback of BTT methods, how to analyze non-uniformly under-sampled BTT signals is a big challenge. In this paper, a novel reconstruction method for non-uniformly under-sampled BTT data is presented. The method is based on the periodically non-uniform sampling theorem. Firstly, a mathematical model of a non-uniform BTT sampling process is built. It can be treated as the sum of certain uniform sample streams. For each stream, an interpolating function is required to prevent aliasing in the reconstructed signal. Secondly, simultaneous equations of all interpolating functions in each sub-band are built and corresponding solutions are ultimately derived to remove unwanted replicas of the original signal caused by the sampling, which may overlay the original signal. In the end, numerical simulations and experiments are carried out to validate the feasibility of the proposed method. The results demonstrate the accuracy of the reconstructed signal depends on the sampling frequency, the blade vibration frequency, the blade vibration bandwidth, the probe static offset and the number of samples. In practice, both types of blade vibration signals can be particularly reconstructed by non-uniform BTT data acquired from only two probes.

## 1. Introduction

High cycle fatigue (HCF) is a common failure mode of high-speed rotating blades in turbo-machinery, such as engine compressor and turbine blades [1]. It often induces blade cracks during the operation, and even catastrophic accidents. Generally speaking, blade vibrations, including synchronous and asynchronous vibrations, are major reasons behind HCF. In particular, when the blade frequency of synchronous vibrations is an integer multiple of the rotating frequency of the bladed-disk, the vibration amplitude of one blade will increase rapidly, which will cause irreversible damage to the blade. Thus nowadays it is urgent to carry out on-line blade vibration monitoring [2–18]. From vibration measurements, stresses induced in the blades may be determined. Action can then be taken to avoid harmful stresses. In this way, it is possible to predict the durability and the life of blades under operating conditions.

Blade tip-timing (BTT) methods have been proposed for non-contact blade vibration monitoring for many years [3–6]. Their outstanding advantages over conventional strain gages are that they are non-contact and can online monitor all-blade vibrations simultaneously. Classical BTT sensors include capacitive, optical-fiber, microwave probes. Among these, optical probes have the highest resolution, so they have been used widely [4,6]. Methods for analyzing BTT data have been developed for a number of applications, such as modal parameter identification of mistuned bladed disks [13,19]. However, since the sampling frequency of BTT methods is determined by the rotation speed and the number of BTT probes, it is always lower than the Nyquist frequency, *i.e.*, twice the maximum frequency of the blade vibration signal. Therefore, the vibration signals collected by BTT methods are always well under-sampled according to the Shannon sampling theorem [20].

Up to now, many studies have been done on monitoring asynchronous vibrations using equally-mounted BTT probes. Zielinski *et al.* [5] used six equally-mounted probes to obtain conclusive vibration frequencies and amplitudes. Garrido *et al.* [8,9] proposed an autoregressive method to obtain modal parameters based on equally-mounted BTT probes. Beauseroy *et al.* [18] proposed a new method to analyze multicomponent blade vibrational signals based on groups of regularly spaced optical sensors. However, in these methods it was difficult to avoid aliasing due to under-sampling. In order to overcome this problem, Bendali *et al.* [13] proposed alternative methods to reconstruct uniformly under-sampled BTT signals for asynchronous vibrations based on the Shannon sampling theorem. Furthermore, Chen *et al.* [14,15] improved the work of Bendali and proposed a novel reconstruction algorithm by combining the Shannon sampling theorem and wavelet packet transformation. In this way, multiple accurate features of asynchronous vibrations are extracted in both the time and frequency domains.

Compared with asynchronous vibrations, synchronous ones are more dangerous to the blades. Unfortunately, the conventional uniform sampling method will be invalid in measuring such vibrations. The reason is that blade vibration frequencies will be an integer multiple of the sampling rate. Assuming that the bladed-disk keeps running at a constant speed, the blade tips will have nominally the same displacement every time they pass the probes. That is to say, the blade tip displacements are essentially repeated over multiple rotations. Therefore, it is nearly impossible to extract true vibration characteristics from the sampling BTT signals. In order to solve this problem, non-equally mounted BTT probes have to be used, instead of equally-mounted ones. The big advantage is that they can be used to measure asynchronous and synchronous blade vibrations simultaneously. However, how to analyze non-uniformly under-sampled BTT signals is a big challenge, including irregular vibration data and frequency aliasing. According to the literature, little work has been done on reconstructing non-uniformly under-sampled BTT signals. To address this knowledge gap this paper will therefore explore a novel method for non-uniformly under-sampled BTT data reconstruction, which should be of great use in blade vibration monitoring.

The remainder of this paper is organized as follows: firstly, the difficulties of monitoring synchronous vibrations are stated in Section 2. In Section 3, a mathematic model of a non-uniform BTT sampling process is built and blade vibration displacements are derived. Then a reconstruction algorithm is proposed in Section 4 based on the periodically non-uniform band-pass sampling theorem. In Section 5 and Section 6, numerical simulations and experimental tests are carried out to validate the feasibility of the proposed method. Finally, some major conclusions are summarized in Section 7.

## 2. Problem Statement

**Figure 1.**
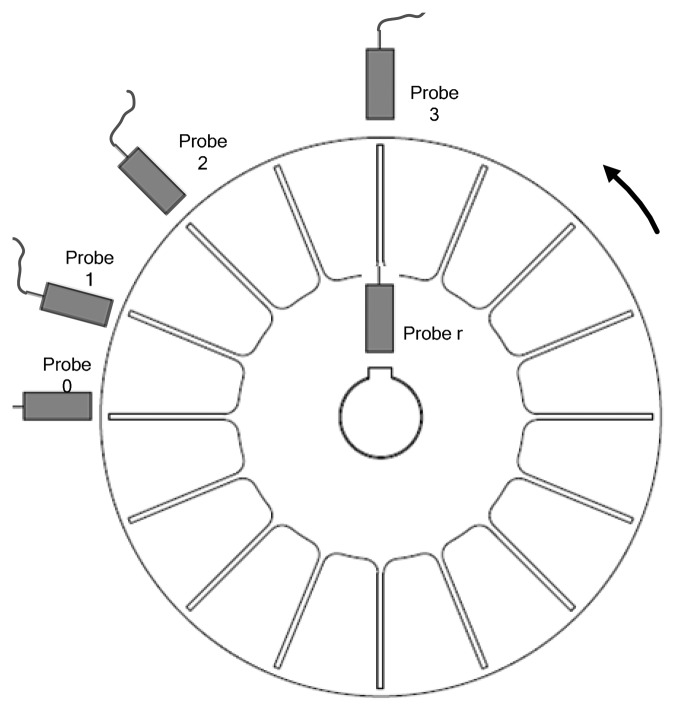
Schematic of the non-uniform BTT sampling.

As shown in Figure 1, optical-fiber probes are embedded non-equally into a stationary casing around a bladed disk. Then the times at which the blade tips pass each probe can be measured. It can be seen that the angles between these sensors are different, so it is a typical non-uniform BTT sampling process.

**Figure 2.**
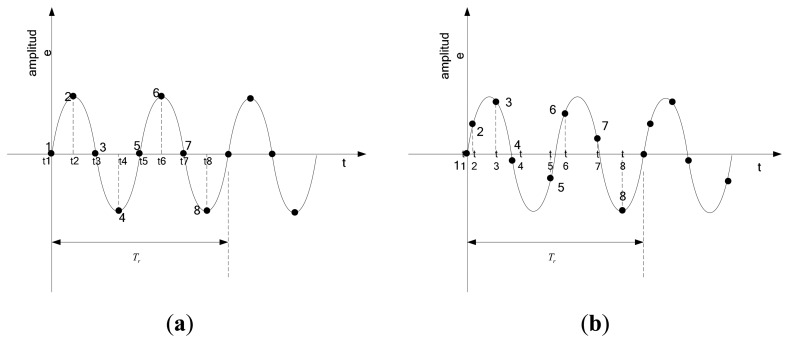
(**a**) The uniform sampling; (**b**) The non-uniform sampling.

Suppose that the rotating period of a rotor is *T_r_* and the blade vibration period is. *T_r_*/2. For a uniform sampling, eight probes are mounted equally to monitor blade vibrations. Each probe associates with a time-series of blade tip displacements, called periodically uniform sample stream. As Figure 2a shows, eight probes are numbered sequentially, starting with one for the first probe. Obviously, the time interval between successive samples is constant, *i.e.*, *t*_3_ −*t*_2_ = *t*_2_ −*t*_1_. However, it is also easily found that one or more probes see the same point on the vibration waveform over multiple rotations, such as probes 1 and probe 5, probes 2 and probe 6, and so on. They record the same displacements every time the blade tips pass them. This phenomenon has two adverse effects. The first one is that probes 5–8 are duplicated for redundancy, which is a waste of probes. The second is that the number of available displacements is reduced from 8 to 4. This makes the analysis of the data become more difficult. In particular, when the blade vibration frequency is an integer multiple of the sampling rate, the number of available displacements can be reduced to 1. This decrease determines that uniform sampling is unsuitable to measure synchronous vibrations.

Conversely, an alternative non-uniform sampling occurs by using eight non-equally mounted probes. As shown in Figure 2b, it is also a sum of eight periodically uniform sample streams. However, in this way, the time interval between successive samples is not constant for all samples, *i.e.*, *t*_3_ −*t*_2_ ≠ *t*_2_ −*t*_1_. Eight points on the vibration waveform are different from each other, so that enough available information can be provided to monitor synchronous vibrations. In fact, it allows measurement of both types of blade vibrations with a reduced number of probes.

Except for the above drawback of uniform sampling, one should attach importance to another fact. That is, in practice, probes are hardly equally spaced in the outer casing due to minimal manufacturing tolerances. Reference [18] has investigated the frequency spectrum of such uniform BTT data. The results indicate that in this way, BTT sampling acts like non-uniform sampling. Thus, it is quite necessary to apply non-uniform BTT sampling to address the drawbacks of uniform sampling.

## 3. Mathematical Model of the Non-Uniform BTT Sampling

### 3.1. Representation of Blade Tip Displacements

As shown in Figure 1, BTT samples are acquired from *I* probes mounted circumferentially around a rotor with *K* blades. An additional probe *r* is mounted in front of the shaft as a reference sensor. There is a white marker line milled on the shaft, so that the reference sensor can measure the once-per-revolution signal. The relative angular position in the casing between probe 0 and probe *i*(0 ≤ *i* < *I*) is denoted as *α_i_*. Without loss of generality, the angular position of probe 0 is set to 0. Similarly, the location of blade *k* is set as *θ_k_*. Since blades are assumed to be equally spaced, one will have *θ_k_* = *θ*_0_ +2*πk*/*K*,*k* ∈ {0,1,…,*K*−1 } where generally *θ*_0_ =0.

The concept of the BTT method is to measure the arrival time of the tip of a vibrating blade as it passes a probe. The expected blade arrival times for a single non-vibrating blade at any probe are determined by the rotation speed, blade tip radius and angular position of the probe. When there are vibrations, any deviations from these expected arrival times indicate blade vibrations with respect to the hub. These deviations are recorded by each probe to calculate a time-series of blade tip displacements and further used to analyze blade vibration characteristics.

Assuming that $t_{i,n}^{k}$ represents the actual arrival time when the blade *k* passes in front of the probe *i* at the *n*-th rotation, and the expected arrival time for a non-vibrating blade is denoted as ${\bar{t}}_{i,n}^{k}$. When the rotating speed *f_r_* is constant, the expected arrival time can be formulated as follows [18]:

(1) $${\bar{t}}_{i,n}^{k}=\frac{1}{2\pi f_{r}}\left(\alpha_{i}+2\pi n-\theta_{k}\right)$$

There is an angular deflection $d\left(t_{i,n}^{k}\right)$ at time $t_{i,n}^{k}$ due to the blade vibration. Thus one will obtain:

(2) $$t_{i,n}^{k}=\frac{1}{2\pi f_{r}}\left(\alpha_{i}+2\pi n-\theta_{k}-d\left(t_{i,n}^{k}\right)\right)$$

Subtracting Equation (2) from Equation (1), one will have:

(3) $$d\left(t_{i,n}^{k}\right)=2\pi f_{r}\left({\bar{t}}_{i,n}^{k}-t_{i,n}^{k}\right)=2\pi f_{r}\Delta t_{i,n}^{k}$$

As shown by Equation (2), the sampling time $t_{i,n}^{k}$ depends on the signal itself. Using $d\left({\bar{t}}_{i,n}^{k}\right)$ instead of $d\left({\bar{t}}_{i,n}^{k}\right)$ is a feasible way to reduce the complexity of following signal processes.

### 3.2. Mathematical Model of the Non-Uniform BTT Sampling

Assuming that a real continued vibration signal of blade *k* is denoted as *r_k_*(*t*). Since the following mathematical model is true for any blade *k*, an arbitrary blade can be considered. Thus, to simplify notations, the index *k* is dropped in the following sections. Based on Equation (3), the blade vibration signal *r*(*t*) will be sampled at the time ${\bar{t}}_{i,n}$. If the rotation speed *f_r_* is constant, the non-uniform BTT sampling function can be formulated as follows:

(4) $$x\left(t\right)=r\left(t\right)\sum_{i=1}^{I-1}\sum_{n=0}^{N-1}\delta \left(t-{\bar{t}}_{i,n}\right)=r\left(t\right)\sum_{i=1}^{I-1}\sum_{n=0}^{N-1}\delta \left(t-\frac{n}{f_{r}}-\frac{\alpha_{i}}{2\pi f_{r}}\right)$$

where *δ*(·) is the Dirac delta function. *x*(*t*) denotes the blade tip displacements, *i.e.*, *x*(*t_i,n_*) = *d*(*t_i,n_*)*R*, where *R* is the distance between the blade tip and the rotating center. Equation (4) is a sum of *I* uniform sample streams. Here a stream associates with a time-series of blade tip displacements recorded by a probe. *a_i_*/2*πf_r_* represents the delay of the *i*-th sample stream. This delay depends strongly on the angular position of probe *i* and the rotation speed. In particular, *a*_0_/2*πf_r_* = 0.

**Figure 3.**
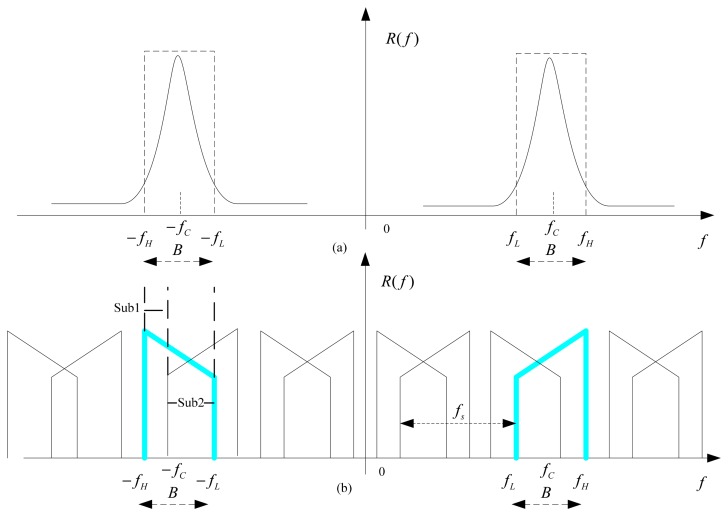
(**a**) The original blade vibration signal; (**b**) The spectrum of one sample stream of the non-uniform sampling.

In practice, the blade vibration is often dominated by a single frequency. The blade response can then be modeled as a single degree of freedom formulation:

(5) $$r\left(t\right)=Ae^{j(2\pi f_{0}t+\varphi )}$$

where *f*_0_ is the dominant frequency. However, the real frequency spectrum of such a blade vibration may have a narrow bandwidth *B*_0_. As shown in Figure 3a, a frequency window with a bandwidth *B* and a central frequency *f_c_* is selected to represent the blade vibration signal, where *f_c_* is an estimated vibration frequency. The guard-band *B_g_* = *B* − *B*_0_ is set to prevent aliasing. The lowest and highest positive frequencies of the window are defined as *f_L_* = *f_c_* −*B*/2 and *f_H_* =*f_c_* + *B*/2, respectively. Therefore the blade tip-timing sampling becomes a band-pass sampling.

## 4. Reconstruction of the Periodically Non-Uniform Band-Pass Sampling

### 4.1. Mathematical Model of the Reconstruction

**Figure 4.**
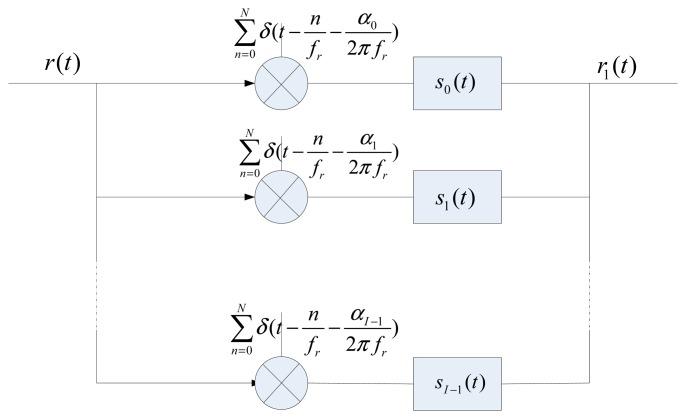
Reconstruction of the periodically non-uniform sampling.

Reference [21] indicated that to reconstruct any sampled signal in the time domain it is necessary to apply an interpolating function to the sampled signal. The non-uniform sampling comprises *I* uniform sample streams. For each stream, an interpolating function is required, as shown in Figure 4. Thus the reconstructed signal can be defined as follows:

(6) $$r_{1}\left(t\right)=\sum_{i=1}^{I-1}\sum_{n=0}^{N-1}r\left(\frac{n}{f_{r}}+\frac{\alpha_{i}}{2\pi f_{r}}\right)S_{i}\left(t-\frac{n}{f_{r}}-\frac{\alpha }{2\pi f_{r}}\right)$$

where *S_i_* (·) is the interpolating function, *r*_1_(*t*)represents the reconstructed signal.

Since it is difficult to directly derive analytical solutions of interpolating functions in the time-domain, one could solve this problem in the frequency-domain. The Fourier transform of *r*_1_(*t*) can be formulated as follows:

(7) $$R_{1}\left(f\right)=\sum_{i=0}^{I-1}f_{r}S_{i}\left(f\right)\sum_{n=-\infty }^{\infty }R\left(f-nf_{r}\right)e^{-jn\alpha_{i}}$$

The frequency spectrum of one sample stream is shown in Figure 3b. The original signal is marked by the blue color. It is easily observed that uniform sampling leads to a periodical replication of the original signal, and a finite number of replicas are intersected in ranges of the original signal, *i.e.*, *f_L_* < *f* < *f*_H_ and − *f_H_* <*f <f*_L_. It must be noted that the aliased contribution is the same for each sample stream except for a different phase shift. To reconstruct the original signal, all unwanted replicas in these ranges must be removed such that *R*_1_(*f*) =*R*(*f*).

To achieve that, the ranges of the original signal are separated into a number of sub-bands. Each sub-band has different intersected parts of replicas, which strongly depend on *B*/*f_r_* [22]. In each sub-band, simultaneous equations are built to ensure that all unwanted replicas sum to zero upon summation of all post-interpolation sample stream. It is assumed that the range of the *jth* sub-band with *N_j_* interacted replicas is denoted as $f_{L}^{j}<f<f_{H}^{j}$. Based on Equation (7), simultaneous equations in this sub-band are built:

(8) $$\{\begin{array}{c} \overset{I-1}{\underset{i=0}{\text{∑}}}f_{r}S_{i,j}\left(f\right)=1\\ \overset{I-1}{\underset{i=0}{\text{∑}}}S_{i,j}\left(f\right)e^{-jn\alpha_{i}}=0,\forall n\in A,n\neq 0 \end{array}$$

where *S_i,j_* represents the *jth* element of *S_i_*(*f*) in the corresponding sub-band. The first equation indicates that the original blade vibration signal in this sub-band should be remained. Other *N_j_* −1 equations are built to remove unwanted replicas, where *A* denotes the index set of unwanted replicas, *i.e.*, $A=\left\{n|\left(f_{H}+nf_{r}\geq f_{H}^{j}\wedge f_{L}+nf_{r}\leq f_{L}^{j}\right)\vee \left(-f_{L}+nf_{r}\geq f_{H}^{j}\wedge -f_{H}+nf_{r}\leq f_{L}^{j}\right)\right\}$.

### 4.2. Blade Vibration Reconstruction by Using Two Probes

Reference [22] has derived a generalization of the analytical solution of *S_i,j_*(*t*). However, it is relatively complicated when *B* > *f_r_*. The calculations of partitioning sub-bands and solving equations are rapidly increasing with the increases of *B*/*f_r_*. It also requires more sample streams to obtain a solution of Equation (8). This requirement conflicts with the limited number of probes in BTT. In order to solve this problem, this paper will propose a non-uniform BTT sampling with only two probes. It is totally enough to reconstruct a blade vibration signal in practice.

In Equation (8), if the number of replicas is greater than the number of probes, *i.e.*, *N_j_* > *I*=2, there will be no solutions for each *S_i,j_*(*f*) since alias-free interpolation requires the solution of more than *I* equations using only *I* variables. In contrary, there will at least one solution for *S_i,j_*(*f*) if *N_j_* ≤ *I* =2. Instantaneously, reference [22] demonstrated that *N_j_* increases with the increases of *B*/*f_r_*, *i.e.*, *N_j_* ∝ *B*/*f_r_*. Especially, when *B*/*f_r_* =1, one will have *N_j_* =2. Hence, if *B* = *f_r_* > *B*_0_, that is, if the rotation speed is more than the narrow bandwidth of the blade vibration signal, it is enough to use two probes to reconstruct a blade vibration signal non-aliasing.

Generally, three or four probes are selected in practice. In the view of this paper, only the case *B* = *f_r_* < *B*_0_ needs more probes simultaneously to reconstruct a signal. Otherwise the blade vibration signal can be reconstructed by using two arbitrary probes. The corresponding reconstruction formula has derived based on the results in [23]:

(9) $$r_{1}\left(t\right)=\sum_{n=-\infty }^{\infty }r\left(\frac{n}{B}\right)S\left(t-\frac{n}{B}\right)+r\left(\frac{n}{B}+\frac{\alpha_{i}}{2\pi B}\right)S\left(-t+\frac{n}{B}+\frac{\alpha_{i}}{2\pi B}\right)$$

where *S* is the interpolation as follows:

(10) $$\begin{array}{l} S\left(t\right)=\frac{\cos\left(2\pi \left(mB-f_{L}\right)t-m\alpha_{i}/2\right)-\cos\left(2\pi f_{L}t-m\alpha_{i}/2\right)}{2\pi Bt\mathrm{Sin}\left(m\alpha_{i}/2\right)}\\ +\frac{\cos\left(2\pi \left(B+f_{L}\right)t-\left(m+1\right)\alpha_{i}/2\right)-\cos\left(2\pi \left(mB-f_{L}\right)t-\left(m+1\right)m\alpha_{i}/2\right)}{2\pi Bt\mathrm{Sin}\left(\left(m+1\right)m\alpha_{i}/2\right)} \end{array}$$

Here *m* = ⌈2*f_L_/B* ⌉. The ceiling operator ⌈ *X* ⌉ denotes the smallest integer not less than *X*. In Equation (10), important constraints should be noticed, *i.e.*, sin(*mα_i_*/2) ≠0 and sin((*m*+1)*α_i_*/2)≠0. Otherwise the interpolation will be meaningless due to the dividing zero. This way, the angular positions of probes should be selected carefully to avoid incorrect reconstructions. In addition, it must be noted that the central frequency *f_c_* and the bandwidth *B* (equal to the rotation speed) of the frequency window should be known *a priori*. Generally, the central frequency *f_c_* can be estimated by FEM methods or modal frequency identification algorithms.

## 5. Numerical Simulations

**Figure 5.**
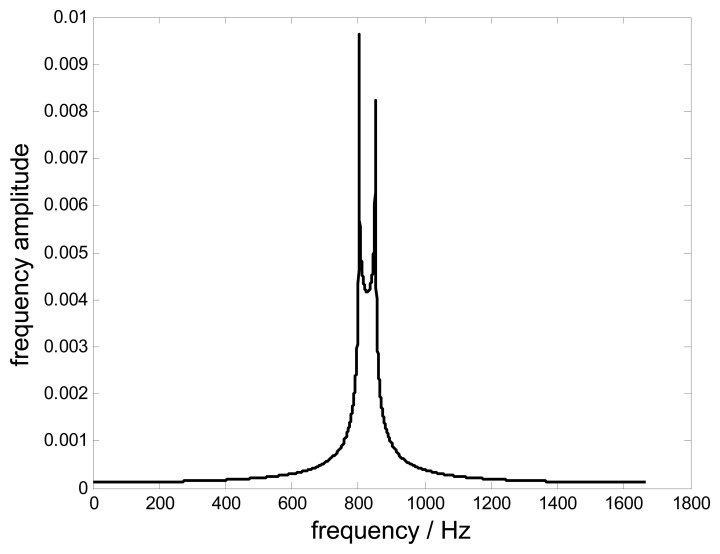
The frequency spectrum of the original signal.

The reconstruction performances of non-uniformly under-sampled BTT data will be evaluated in terms of the sampling frequency, the blade vibration frequency, the blade vibration bandwidth, the probe static offset and the number of samples. Since the blade vibration is a narrowband signal, in order to provide more insight, the vibration signal in these simulations is simply replaced by a typical band-pass signal, *i.e.*, *f*(*t*) = *c*(*B*_0_*t*)sin(2*πf_c_t*)[16]. All conclusions are also established for real blade vibrations. With the setting of *B*_0_ =50 Hz, *f_c_*=827 Hz Figure 5 shows the frequency spectrum of this original signal. The related simulation parameters of BTT are shown in Table 1. In this case, the individual sampling rate will be equal to the rotating speed, *i.e.*, *f_r_* = 5000 *n* / min 83.3Hz.

**Figure 6.**
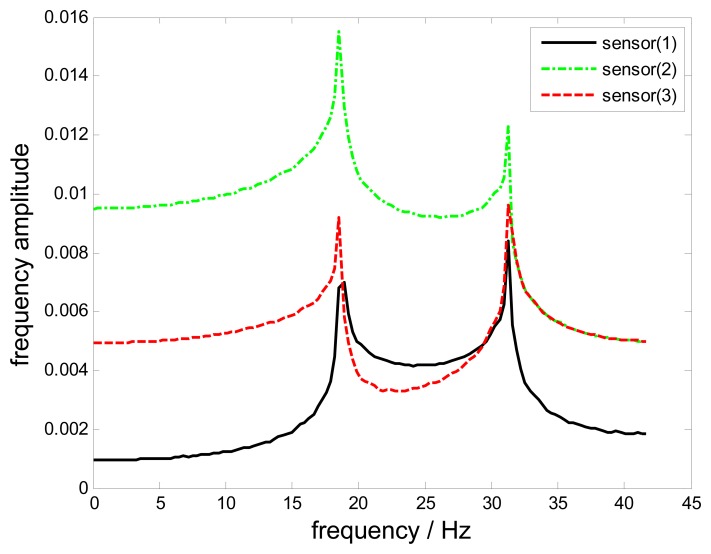
The frequency spectra of sample streams with *f_r_* =83.3 Hz.

The aliasing of under-sampling is evaluated first. The frequency spectra of all three uniform sample streams with *f_r_* = 83.3Hz are shown in Figure 6. The curves of various probes are the superposition of two replicas of the original signal with different phase shift, which are significantly different due to aliasing.

### 5.1. Definition of the Reconstruction Error

**Figure 7.**
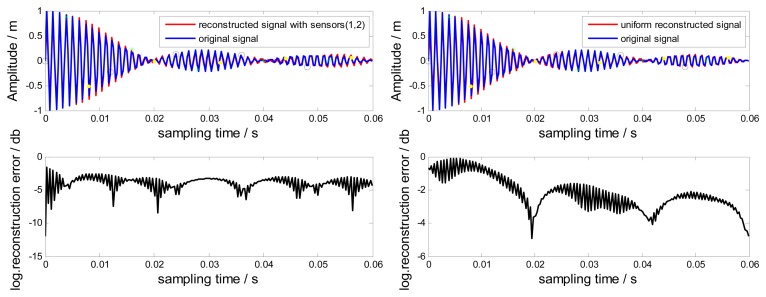
Reconstructed signals and local logarithmic reconstruction errors.

In this section, no-uniformly under-sampled BTT signals from Probes (1, 2) are used to reconstruct the original signal. Uniform BTT signals from probes (1, 2, 3) are also used to reconstruct the original signal based on the Shannon sampling theorem [14,23]. Compared results between reconstructed signals and the original signal are shown in Figure 7. The corresponding local logarithmic reconstruction error [14] is also shown in Figure 7, defined by:

(11) $$e\left(x\right)=\log\left(\left|r\left(t\right)-r_{1}\left(t\right)\right|\right)$$

It can be easily found that the reconstructed signals by Probes (1, 2) are proximate to the original signal. The corresponding local logarithmic reconstruction error is relatively small and uniform in comparison with that of the uniform reconstruction. Since interpolations require many terms of samples to be evaluated, using a finite number of samples will affect the accuracy of the reconstructed signal, especially the local signals close to the start and end of samples. The uniform reconstruction suffers more loss since the kernel function sin *c*(●) used by the Shannon sampling theorem is known to decay very slowly. However, a promising way out is to apply a time-limited reconstruction kernel function [13,16] to replace the sin *c*(●)function so that only few samples have to be involved.

**Figure 8.**
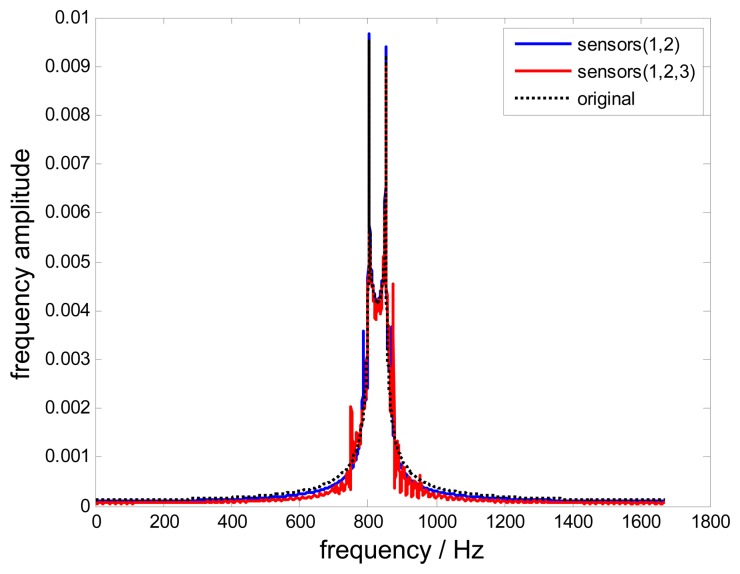
Frequency spectra of reconstructed signals.

Figure 8 shows frequency spectra of reconstructed signals and the original signal. The non-uniform reconstructed signal is almost as the same with the original signal. However, a mutation still occurs in the boundaries of the selected frequency window.

### 5.2. Reconstruction Error Affected by the Bandwidth B_0_

**Figure 9.**
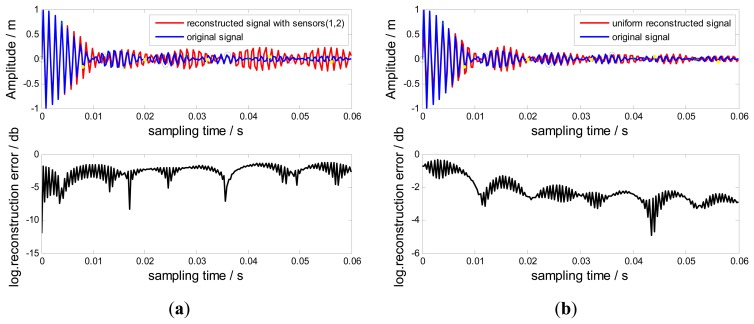
Reconstructed signals and the original signal with *B_0_* = 100 Hz for (**a**) the non-uniform sampling and (**b**) the uniform sampling.

**Figure 10.**
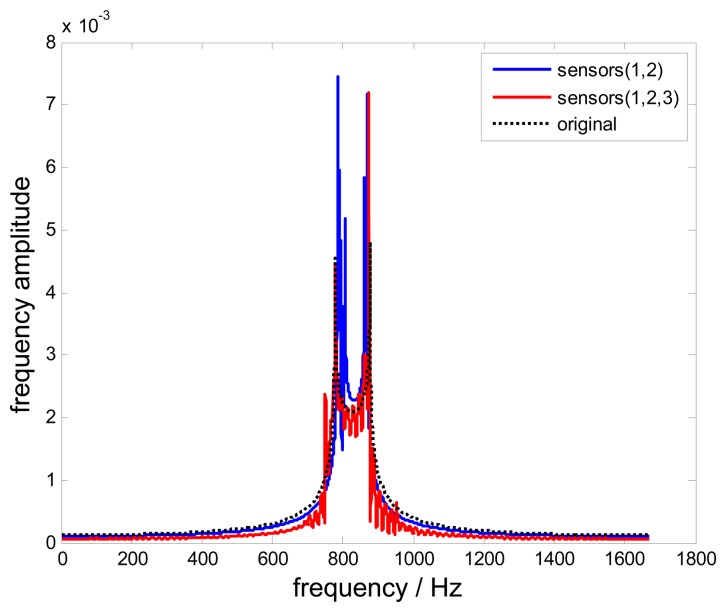
Frequency spectra of reconstructed signals and the original signal with *B_0_* = 100 Hz.

In this section, simulations are done to estimate the reconstruction error affected by the bandwidth *B*_0_. In Equation (9), the bandwidth *B* of the frequency window depends on the sampling rate *f_r_*, *i.e.*,*B* =*f_r_* =83.3Hz. Setting *B*_0_= Hz > *B*, the corresponding reconstructed signals in the time-domain and frequency-domain are shown in Figures 9 and 10, respectively.

Obviously, there are significant differences between the original signal and the non-uniform reconstructed signal. The reconstruction is aliasing. However, the reconstruction error of the uniform reconstructed signal is smaller than that of the non-uniform one. This is because the uniform sampling using three probes has a wider bandwidth *B*_1_ of the frequency window, where *B*_1_> *B*. In order to obtain a non-aliasing reconstructed signal for this case, there are two feasible ways. First, one can increase the rotating speed to increase the bandwidth *B*. Second, if increasing the rotation speed is not allowed since it will change the blade vibration characteristics, probes could be added to ensure there are solutions for *S_i,j_*(*f*) in Equation (8) when *B* > *f*_r_.

### 5.3. Reconstruction Error Affected by the Vibration Frequency f_0_

**Figure 11.**
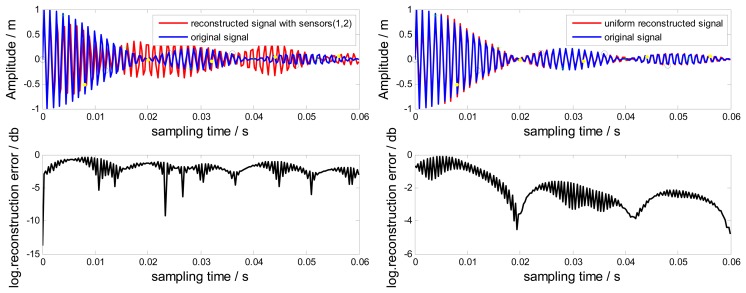
Reconstructed signals and the original signal with ${\bar{f}}_{c}=800\;\text{Hz}$.

**Figure 12.**
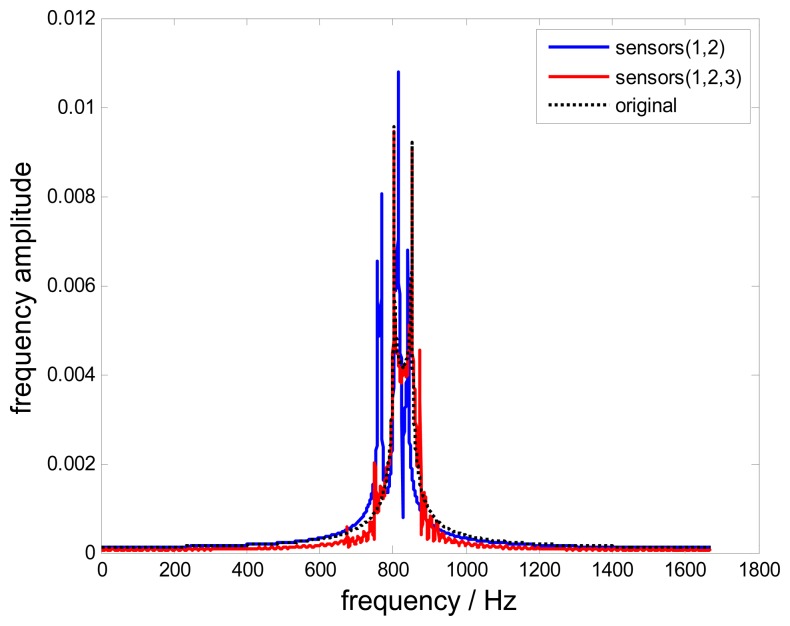
Frequency spectra of reconstructed signals with ${\bar{f}}_{c}=800\;\text{Hz}$.

The blade vibration frequency *f*_0_ is an important parameter reflecting the health of a blade. It is often estimated by various methods [7,8,11]. However, the uncertainties between estimated value ${\bar{f}}_{0}$ and the original one *f*_0_ may cause a severe reconstruction error. The blade vibration frequency is set as *f*_0_ = 827Hz. The estimated frequency is assumed as ${\bar{f}}_{0}=800\;\text{Hz}$, so that the central frequency is selected as $f_{c}={\bar{f}}_{0}=800\;\text{Hz}$. The uniform and non-uniform reconstructed signals are shown in Figure 11. The non-uniform reconstruction error is relatively large, since the frequency spectrum of this signal has shifted from the original position as shown in Figure 12. It has become a superposition of a replica and the original signal. In contrast, the uniform reconstruction has little changes in comparison with that shown in Figure 7b. In this case, the bandwidth *B*_1_ of the frequency window of the uniform reconstruction is wide enough, such that the original signal still locates at the window range $\left[{\bar{f}}_{c}-B_{1}/2,{\bar{f}}_{c}+B_{1}/2\right]$ as shown in Figure 12. For non-uniform sampling, an adjustment of the estimated frequency should be proposed to reduce the reconstruction error, which will be the next work.

### 5.4. Reconstruction Error Affected by the Number of Samples

**Figure 13.**
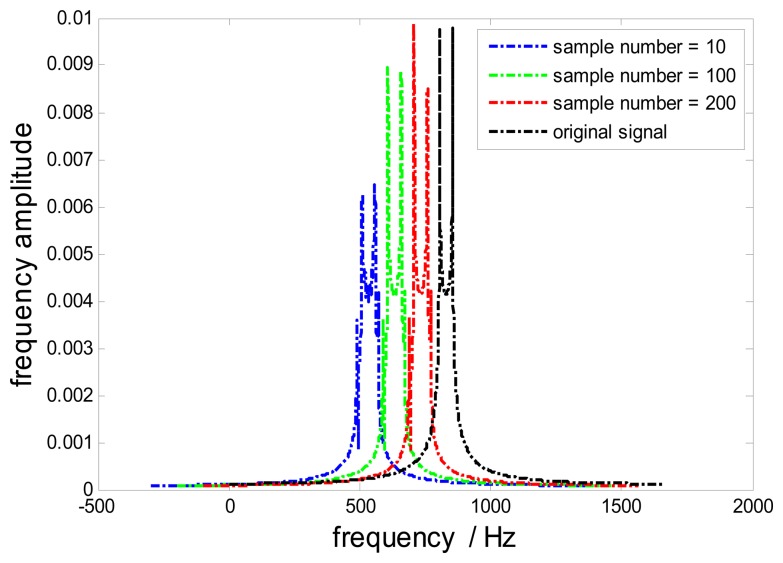
Frequency spectra of reconstructed signals with various numbers of samples.

In practice, a reconstruction error will occur since only a finite number of samples are used to reconstruct a signal based on Equation (9). In order to reduce this error, frequency spectra of reconstructed signals with various numbers of samples are investigated in Figure 13. To identify different curves, each curve is shifted by 100 Hz from each other. It is easily found that with the increases of the number of samples, the reconstructed signal is more approximate to the original signal. Generally, 200 samples of each uniform sample stream are enough to reconstruct the original signal approximately.

### 5.5. Reconstruction Error Affected by the Probe Static Offset

**Figure 14.**
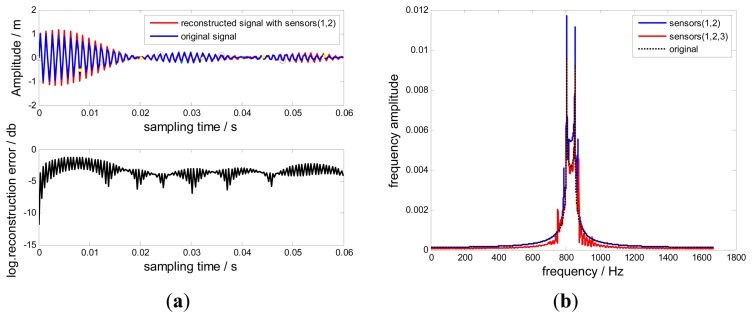
Reconstructed signals with the probes offset in (**a**) time-domain and (**b**) frequency-domain.

The accurate reconstruction also depends on the accuracy of angular positions of probes. Setting the probe static offset Δ*α_i_* =2°, frequency spectra of reconstructed signals are shown in Figure 14. The significant reconstruction error indicates the probe static offset due to manufacturing tolerances should be kept minor.

### 5.6. Summary

In this section, the aliasing of under-sampling has been evaluated. Then the reconstruction performances are evaluated in terms of the blade vibration frequency, the blade vibration bandwidth, the probe static offset and the number of samples. The results show that the proposed method is feasible for reconstructing original signals by using more than 200 samples of each probe. Additionally, assuring that the original blade vibration frequency *f*_0_ locates within the specified frequency window [*f_L_*, *f_H_*] is very important to decrease reconstruction errors, and probe static offsets should be kept small too.

## 6. Experiments

**Figure 15.**
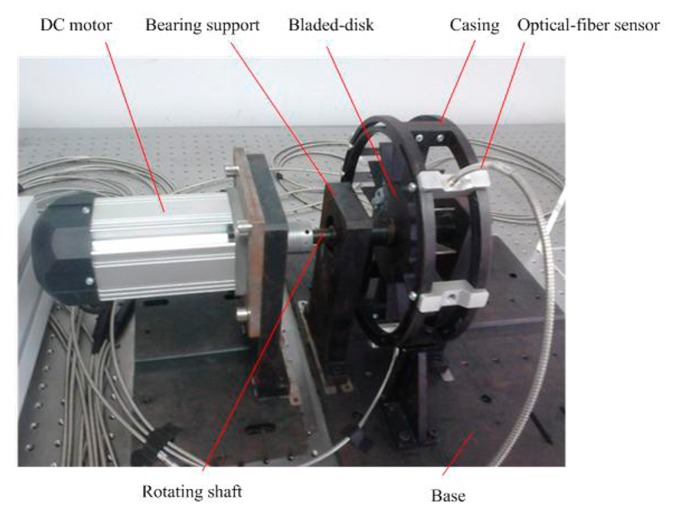
The test rig.

As shown in Figure 15, an experimental set-up is built to validate the feasibility of the proposed method. In order to compare with the uniform sampling, three optical-fiber probes are embedded equally in the circular bracket to sample arrival times. Using two arbitrary probes forms a non-uniform sampling. The additional optical-fiber probe is placed close to the rotating shaft for sampling reference time.

**Figure 16.**
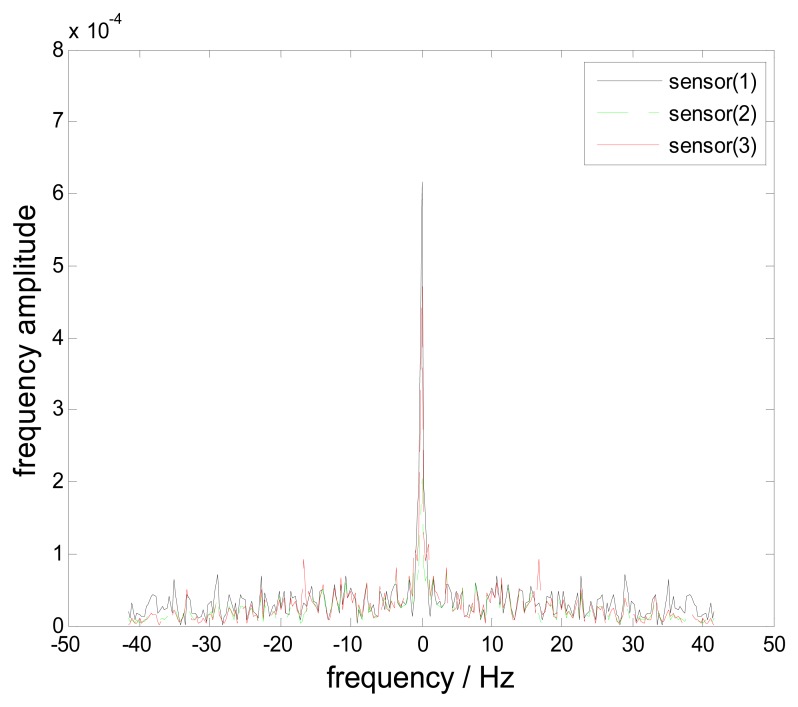
The frequency spectra of sample streams.

Other detailed experimental parameters are shown in Table 1. The blade vibration frequency is estimated by FEM simulations, *i.e*, ${\bar{f}}_{0}=827\;\text{Hz}$, such that the central frequency is selected as $f_{c}={\bar{f}}_{0}$. A long time test is done to collect at least 3000 samples. Four-channel time impulse signals are collected and all-blade tip displacements are calculated based on Equation (3). Here vibration signals of Blade 0 are considered. The frequency spectra of all three uniform sample streams are shown in Figure 16. The peaks of the spectra are located at $\bar{f}=0$. Based on the sampling theorem, the blade vibration frequency can be inferred as $f_{0}=nf_{r}+\bar{f}=nf_{r}$.

**Figure 17.**
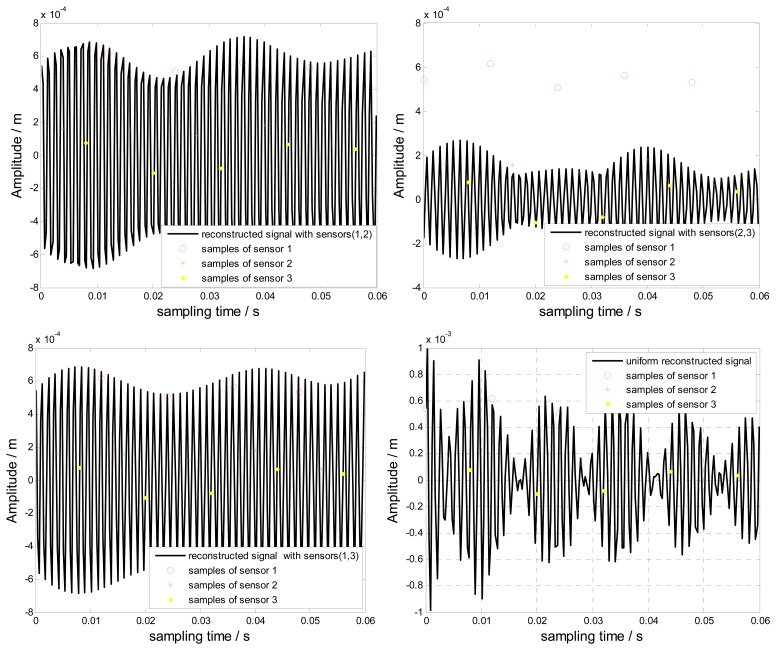
Reconstructed Signals.

The reconstructed signals from various probes are shown in Figure 17. The reconstructed signal curves using probes (1, 2) and probes (1, 3) are similar. However, the curve using probes (2, 3) is not so precise. The measurement noise and probe static offsets forced on these probes could induce significant reconstruction errors. These errors have been also investigated in [21,24]. The results in these papers could be further used to optimize the reconstruction process. We will also focus on decreasing these harmful impacts in our future work.

**Figure 18.**
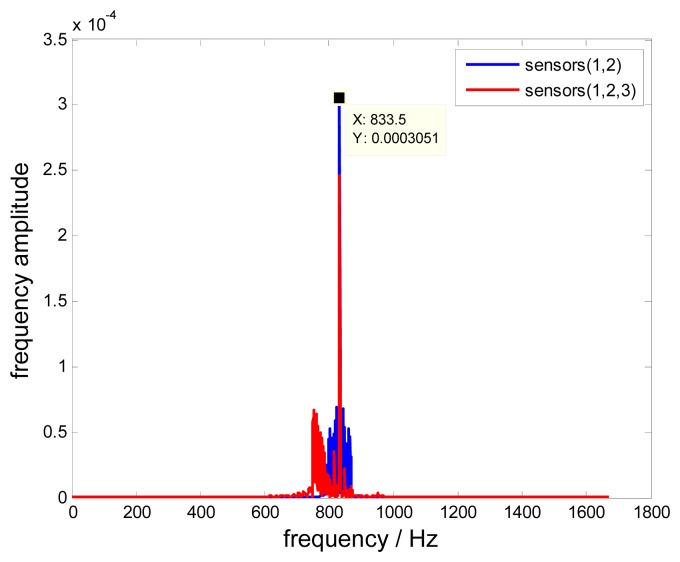
Frequency spectra of reconstructed signals.

Additionally, the uniform reconstructed signal is also different from the non-uniform one in the time domain as shown in Figure 17. Nevertheless, the frequency spectra in Figure 18 show that the blade vibration frequency *f*_0_=833.5Hz can be precisely detected from these two different reconstructed signals, which validates the feasibility of the proposed method. However, it must be noted that different notable frequencies exist nearby 833.5 Hz, which causes reconstructed signals to be distorted. These differences are dominated by the measurement noise propagated in these two different approaches, therefore, to improve the signal-to-noise ratio and optimizing the reconstruction process is very important, which is one key point of our future work.

## 7. Conclusions

The blade tip-timing method has become an important non-contact way to monitor blade vibrations online. In order to obtain accurate vibration characteristics, two key points are important. First, it is well known that under-sampling is an intrinsic drawback of BTT methods. Thus a signal reconstruction based on BTT data is required to solve this bottleneck problem. Second, non-equally mounted probes have been used to measure synchronous and asynchronous vibrations instantaneously. However, nowadays little work has been done on reconstructing non-uniformly under-sampled BTT signals. In this paper, a novel reconstruction of non-uniformly under-sampled BTT data is proposed based on the periodically non-uniform sampling theorem. Firstly, all-blade vibration displacements using optical-fiber probes are calculated based on BTT methods. Then a mathematical model of a non-uniform BTT sampling process is built. It can be treated as the sum of certain uniform sample streams. For each stream, an interpolating function is required to prevent aliasing in the reconstructed signal. To achieve that, sub-bands are defined in the range of the original signal. Each sub-band has different intersected parts of replicas of the original signal. Next, it builds simultaneous equations of all interpolating functions in each sub-band and ultimately derives the solutions to remove unwanted replicas of the original signal caused by the sampling. In the end, numerical simulations and experiments are done to validate the feasibility of the proposed method. The main results are summarized as follows:

1. A non-aliasing reconstruction of non-uniformly under-sampled BTT data based on the periodically non-uniform sampling theorem is presented.
2. The accuracy of the reconstructed vibration signal depends on the sampling frequency (the rotation speed), the blade vibration frequency, the blade vibration bandwidth, the probe static offset and the number of samples.
3. In practice, a blade vibration signal can be particularly reconstructed by non-uniformly under-sampled BTT data acquired from only two probes if the blade vibration frequency of the blade is known in advance.

## Figures and Tables

**Table 1.** Experimental Setting.

**Parameters**	**Properties**
Material of the bladed-disk	Type 45 steel
The number of blades	16
The length of each blade	45 mm
The width of each blade	20 mm
The thickness of each blade	2 mm
The distance of the blade tip to the center	95 mm
The rotating speed	5000 rpm
The angular position of probe 1	0°
The angular position of probe 2	120°
The angular position of probe 3	240°
